# Editorial: Exploring the role of immune cells and cell therapy in liver cancer

**DOI:** 10.3389/fimmu.2025.1752606

**Published:** 2025-12-15

**Authors:** Wencheng Zhang, Yan-Ru Lou, Yue Wang, Xicheng Wang, Zhiying He

**Affiliations:** 1 Institute for Regenerative Medicine, Medical Innovation Center and State Key Laboratory of Cardiovascular Diseases, Shanghai East Hospital, School of Life Sciences and Technology, Tongji University, Shanghai, China; 2 Shanghai Engineering Research Center of Stem Cells Translational Medicine, Shanghai, China; 3 Shanghai Institute of Stem Cell Research and Clinical Translation, Shanghai, China; 4 Shanghai Children’s Hospital, School of Medicine, Shanghai Jiao Tong University, Shanghai, China; 5 Research Center of Developmental Biology Department of Histology and Embryology College of Basic Medicine, Second Military Medical University, Shanghai, China; 6 Shanghai Key Laboratory of Cell Engineering Second Military Medical University, Shanghai, China

**Keywords:** chimeric antigen receptor (CAR) T-cell therapy, immunotherapy, liver cancer (LC), natural killer cells (NK cells), programmed cell death protein-1 (PD-1), tumor microenvironment (TME), tumor organoids

Hepatocellular carcinoma (HCC) is one of the most common cancers worldwide, underscoring the urgent need for improved diagnosis and treatment. One of the most critical aspect for the complexity of the HCC is its tumor microenvironment (TME), a complex structure composed of various immune cells, cancer-associated fibroblasts, endothelial cells, and other tissue-resident cells., The TME plays a crucial role in the occurrence, development, invasion, infiltration, metastasis, spread, and growth of tumors. Understanding the complex interactions between tumor cells and the TME is not only a prerequisite for the rational development of effective anti-tumor therapies but also key to targeted therapy and effective drug delivery. This Research Topic of *Frontiers in Immunology* explores the latest advances in developing cell or immunotherapies with stable quality and significant efficacy in the context of tumor and immune microenvironment. These innovations offer promising strategies for treating liver tumors that are currently untreatable in clinical practice and provide useful research and translational directions for cell therapy against other solid tumors.

Immunotherapy for liver cancer can be broadly categorized into traditional targeted therapy and novel immune cell therapy. Regarding traditional treatments, Dong et al. conducted a retrospective real-world study on the poor prognosis and ineffectiveness of existing treatments for advanced intrahepatic cholangiocarcinoma (ICC). Analysis of data from 95 ICC patients revealed that chemotherapy combined with lenvatinib and programmed cell death protein-1 (PD-1) inhibitors was significantly effective and well-tolerated, representing a potentially better treatment option for advanced unresectable intrahepatic cholangiocarcinoma. However, this conclusion still requires validation through larger-scale prospective cohort studies. Chen et al. also conducted a systemic meta-analysis to examine the efficacy and safety of transarterial chemoembolization (TACE) plus lenvatinib with or without PD-1 inhibitors (TLP group) compared with TACE + lenvatinib (TL group) for unresectable hepatocellular carcinoma (uHCC). And their result concluded that the TLP group had better efficacy for uHCC than that of the TL group, with an acceptable safety profile.

Beyond classic sites like PD-1, many studies have focused on novel targets. Chen et al. elucidated the crucial role of caspase-8 in the development, progression, and drug resistance of HCC, and explored the prospects of targeting caspase-8 as a treatment for HCC. However, the authors also noted that the regulatory role of caspase-8 in the complex TME of HCC is not fully understood. Furthermore, the clinical translation of this approach faces significant technical hurdles, including the lack of highly specific and selective caspase-8 activators and inhibitors, as well as the lack of effective delivery to tumor tissues and the ability to penetrate the vascularized TME of HCC.

As of the immune cell therapy, Zhang et al. reviewed the application of cell therapy in HCC, covering different cell types, their effective mechanisms, the latest advances in clinical trials, and current challenges. Their work provides useful insights for future research and clinical applications in the treatment of HCC. To date, the chimeric antigen receptor (CAR) T-cell therapy has been known as a key advancement in cancer treatment, but many challenges remain in using CAR-T cells to treat solid tumors such as HCC. Key issues that still need to be addressed include improving T-cell migration, combating the immunosuppressive TME, and enhancing safety. A mini-review by Zhou et al. summarizes the latest research findings and clinical progress of CAR-T cell therapy in the treatment of HCC, providing a comprehensive overview of the current status, challenges, and future prospects of CAR-T cell immunotherapy in HCC treatment.

The development of alternative immune cell therapy is based on clarifying the composition and dynamic changes in immune cell populations in disease states compared to healthy condition. Natural killer cells (NK cells) play a role in both innate and adaptive immune responses. Multiple studies have confirmed that NK cell phenotype and specific functions are influenced by the microenvironment. Hepatic NK cells undergo functional and phenotypic changes during liver cancer progression, affecting disease prognosis. To explore the effects of immune cells or immune signaling pathways on the behavior of tumor cells and tumor-initiating cells, Antonia Paul et al. established a 37-color flow cytometry method incorporating 41 markers to perform in-depth phenotypic analysis of human peripheral and hepatic NK cells. This high-parameter, high-resolution detection platform provides a key tool for in-depth analysis of different NK cell subsets in peripheral blood and liver under healthy and disease states.

Other than NK cells, dendritic cells (DCs), a diverse class of professional antigen-presenting cells, also holds promise as an effective strategy to improve the efficacy of anti-tumor immunotherapy and enhance tolerance to autoantigens in autoimmune diseases. Wang et al. found that oral administration of a Toll-like 2 receptor (TLR2)- activating lactic acid-producing probiotics (LAP) can effectively and significantly induce the accumulation of regulatory dendritic cells (rDCs) in the liver of mice. This accumulation inhibited the function of cytotoxic T cells and alleviated diethylnitrosamine (DEN)-induced liver injury, fibrosis, and tumorigenesis. Considering the role of LAP in stimulating regulatory DCs, this therapeutic strategy may also have good clinical application value in the prevention or treatment of autoimmune diseases (arthritis and asthma), inflammatory bowel disease, and alcoholic or non-alcoholic chronic liver disease.

Of course, any immunotherapy, while establishing the effectiveness of drugs and cells, must also acknowledge the unique immune microenvironment of the liver. The liver’s immune microenvironment not only inhibits the efficacy of immunotherapeutic drugs but also creates a barrier, leading to drug resistance and reducing the overall effectiveness of treatment. Liu et al. summarized recent research progress on the immune profile of liver cancer, pointing out that future treatment strategies include combining immunotherapy with other therapies, utilizing targeted therapies to modulate the immune microenvironment, and developing novel drugs that can bypass or counteract liver inhibitory mechanisms, thereby improving the treatment outcomes of liver cancer.

In addition to focusing on the progress of immunotherapy for HCC, this Research Topic also summarizes models for liver cancer research and prognostic assessment. Song et al. summarized the current research and treatment progress for fibrolamellar carcinoma (FLC), a rare tumor. They particularly elucidated the crucial role of the interaction between FLC epithelial cells, endothelial progenitor cells, stellate cells, and the host’s immune microenvironment in the construction of FLC organoids. The review emphasized the key role of the interaction between tumor cells and multiple cell types within the TME in tumor organoid construction, and the concepts mentioned in this review also apply to the construction of organoids for HCC and cholangiocarcinoma.

Lastly, as a direct manifestation of the role of the tumor immune microenvironment in tumor recurrence and metastasis, microvascular invasion (MVI) is an independent risk factor for recurrence and metastasis of HCC, highly associated with poor prognosis. Mu et al. identified characteristic genes of MVI and constructed a novel prognostic prediction model for HCC using spatial transcriptome sequencing. This model suggests an increased proportion of macrophages in high-risk patients, indicating that HCC tumor cells may promote HCC metastasis through macrophage cell interactions via activating “migration inhibitory factor (MIF)-CD74” signaling. This study not only provides an assessment model for the prognosis of patients with HCC but also has important clinical significance in differentiating patient types and selecting appropriate treatment options.

In summary, this Research Topic provides a systematic and comprehensive overview of the current progress, breakthroughs, existing problems, and solutions in traditional drug immunotherapy and cell therapy for HCC. It underscores the development of sequencing for targeted therapy and cell therapy, as well as combined systemic therapies, for solid tumors such as HCC.

